# Editorial: Nutritional quality formation and maintenance of horticultural crops

**DOI:** 10.3389/fpls.2022.1005338

**Published:** 2022-08-11

**Authors:** Tong Chen, Jingjing Xing, Mohamed El-Mogy, Yuhua Wang

**Affiliations:** ^1^Key Laboratory of Plant Resources, Institute of Botany, Chinese Academy of Sciences, Beijing, China; ^2^State Key Laboratory of Crop Stress Adaptation and Improvement, School of Life Sciences, Henan University, Kaifeng, China; ^3^Vegetable Crops Department, Faculty of Agriculture, Cairo University, Giza, Egypt; ^4^College of Horticulture, Nanjing Agricultural University, Nanjing, China

**Keywords:** horticultural crops, quality trait, defense response, postharvest, fruits, vegetables

Horticultural crops bring about numerous health-promoting benefits, e.g., dietary fibers, vitamins, antioxidants, and other secondary metabolites, thereby being favored by consumers worldwide (Chen et al., 2021). The formation and maintenance of nutritional quality depends on intrinsic characteristics of horticultural crops and environmental conditions, which directly determine disease resistance, final yields and commodity (Szymański et al., 2020; Xu et al., 2022). Therefore, additional efforts have been made to dissect the mechanisms regulating quality formation and maintenance during the past several decades.

In an attempt to address and update our knowledge toward nutritional quality for fruits, vegetables, fresh flowers and tea, we have organized this Research Topic on “*Nutritional quality formation and maintenance of horticultural crops*.” With an overwhelming response from several leaders in the field of stress signaling, a total of five articles (two reviews and three original articles) have been published in this Research Topic. Mostafa et al. contributed a review article “*Floral scents and fruit aromas: Functions, compositions, biosynthesis, and regulation*.” This review firstly introduces the composition of floral scents and fruit aromas in some representative flowers and fruits (such as rose, orchid, tulip, lily, banana, apple, strawberry, citrus, and others). Moreover, the authors further compared the biosynthetic pathways and metabolic activities of terpenes, phenylpropanoid/benzenoid, fatty acid derivatives, amino acid derivatives, major substances contributing to floral scents, and fruit aromas. The regulations on the emission of these volatile organic contents (VOCs) at transcriptional and epigenetic levels were also illustrated, with emphasis on the functions of major transcription factors. Finally, the authors envisage some unsolved issues related to floral scents and fruit aromas and point out directions of future efforts. This review collectively narrates floral scents and fruit aromas from their production to metabolism and genetic regulations, providing valuable datasets for further attempts to improve quality traits by metabolic engineering.

Another review “*The Akebia genus as a novel forest crop: A review of its genetic resources, nutritional components, biosynthesis, and biological studies*” is contributed by Huang et al. Having been used as Chinese herbal medicine for a long history, *Akebia* species have the potential to be developed as forest crops with high nutritional and economic value for their delicious taste and abundant nutrients. Huang et al., elaboratively describe major biological and ecological characteristics of the *Akebia* species, further introduce major phytochemical components (mainly sugars and triterpenoids), nutritional traits and main uses. As the chromosome-level genome sequence of *A. trifoliata* is available and the tissue culture attempt is also making progresses, further genetic manipulation and engineering may greatly accelerate the exploration of *Akebia* species as economically important medicinal and edible plants.

The three research articles focus on transcriptomic and metabolomic variations in tea plants (*Camellia sinensis*) as well as the relationship between iridoid glycoside accumulation and DNA methylation in *Rehmannia glutinosa*. Yue et al. reported the functions of Golden2, ARR-B, Psr1 (GARP) family members in *C. sinensis*. The authors examined phylogenetic relationships, gene structures, chromosomal locations, conserved motifs, and regulatory *cis*-acting elements for these *CsGARP* genes. The data for their subcellular localization, tissue-specific and condition-specific expression patterns may provide certain references for further studies on the functions of *CsGARP* genes in tea plant. Another study contributed by Xu et al. investigates transcriptomic and metabolomic variations in response to nitrogen deprivation and resupply in tea plant (*Camellia sinensis*) roots. Nitrogen deficiency triggered significant decreases in certain amino acids, polyphenols, and caffeine, while nitrogen resupply restored the contents of amino acids and polyphenols. These variations were attributed to the activation of flavonoids-related pathways as well as activities of a bulk of key genes and transcriptional factors. These results may provide certain guidance on the improvement of nitrogen use efficiency to facilitate quality formation in tea plants.

Dong et al. performed RNA-seq analysis and further identified a total of 357 unigenes involved in iridoid glycoside biosynthesis in *Rehmannia glutinosa*. The treatment with 5-azacytidine (5-azaC), a DNA transferase inhibitor, generally upregulated the expression of *DXS, DXR, GPPS, G10H*, and *10HGO* (key genes involved in iridoid glycoside synthesis in *R. glutinosa*) in roots and leaves, which corresponded to a decreased methylation modification at the genomic level. Coincidently, iridoid glycoside accumulation also increased. These results well-correlated DNA methylation, transcriptional activities of key genes and iridoid glycoside accumulation in *R. glutinosa*.

In general, the reviews and original researches in this Research Topic emphasized the important contribution and underlying mechanisms for nutritional quality formation and maintenance of horticultural crops. Researchers have made efforts to explore the machinery responsible for nutritional trait formation, dissect the regulatory factors on biosynthesis, metabolism and catabolism of key metabolites at the physiological, genetic and epigenetic levels. We have to admit that many aspects of nutritional quality and relevant regulatory mechanisms are not fully covered due to short of manuscripts and limitations in the scope of knowledge. However, attempts for elucidating different aspects of nutritional quality of horticultural crops also put forward some unsolved questions such as what are similarities and differences between floral scents and fruit aromas? how is iridoid glycoside accumulation regulated by DNA methylation? In addition, one of the major challenges we have to confronted with is how horticultural plants can be genetically manipulated to improve their nutritional quality without growth penalty, while CRISPR-Cas9 technology may substantially facilitate in-depth explorations in these fields (Xu et al., 2022). Indeed, the state-of-art multidisciplinary techniques of omics, biochemistry, physiology and molecular biology may undoubtedly accelerate the genetic and metabolic engineering of nutritional traits for horticultural crops (Zhu et al., 2018).

## Author contributions

TC wrote the article. TC, JX, ME-M, and YW revised the manuscript. All authors contributed to the article and approved the submitted version.

## Funding

This work was supported by the grants from National Natural Science Foundation of China (32072637) and Beijing Municipal Natural Science Foundation (6212025).

## Conflict of interest

The authors declare that the research was conducted in the absence of any commercial or financial relationships that could be construed as a potential conflict of interest.

## Publisher's note

All claims expressed in this article are solely those of the authors and do not necessarily represent those of their affiliated organizations, or those of the publisher, the editors and the reviewers. Any product that may be evaluated in this article, or claim that may be made by its manufacturer, is not guaranteed or endorsed by the publisher.
